# Waterborne exposure to BPS causes thyroid endocrine disruption in zebrafish larvae

**DOI:** 10.1371/journal.pone.0176927

**Published:** 2017-05-03

**Authors:** Dan-hua Zhang, En-xiang Zhou, Zhu-lin Yang

**Affiliations:** 1 Department of General Surgery, The Second Xiangya Hospital, Central South University, Changsha, Hunan, China; 2 Research Laboratory of Hepatobiliary Diseases, The Second Xiangya Hospital, Central South University, Changsha, Hunan, China; Deakin School of Medicine, AUSTRALIA

## Abstract

Bisphenol S (BPS) is widely used as a raw material in industry, resulting in its ubiquitous distribution in natural environment, including the aqueous environment. However, the effect of BPS on the thyroid endocrine system is largely unknown. In this study, zebrafish (*Danio rerio*) embryos were exposed to BPS at 1, 3, 10, and 30 μg/L, from 2 h post-fertilization (hpf) to 168hpf. Bioconcentration of BPS and whole-body thyroid hormones (THs), thyroid-stimulating hormone (TSH) concentrations as well as transcriptional profiling of key genes related to the hypothalamic-pituitary-thyroid (HPT) axis were examined. Chemical analysis indicated that BPS was accumulated in zebrafish larvae. Thyroxine (T4) and triiodothyronine (T3) levels were significantly decreased at ≥ 10 and 30 μg/L of BPS, respectively. However, TSH concentration was significantly induced in the 10 and 30 μg/L BPS-treated groups. After exposure to BPS, the mRNA expression of corticotrophin releasing hormone (*crh*) and thyroglobulin (*tg*) genes were up-regulated at ≥10 μg/L of BPS, in a dose-response manner. The transcription of genes involved in thyroid development (*pax8*) and synthesis (sodium/iodide symporter, *slc5a5*) were also significantly increased in the 30 μg/L of BPS treatment group. Moreover, exposure to 10 μg/L or higher concentration of BPS significantly up-regulated genes related to thyroid hormone metabolism (deiodinases, *dio1*, *dio2* and uridinediphosphate glucoronosyltransferases, *ugt1ab*), which might be responsible for the altered THs levels. However, the transcript of transthyretin (*ttr*) was significantly down-regulated at ≥ 3 μg/L of BPS, while the mRNA levels of thyroid hormone receptors (*trα* and *trβ*) and *dio3* remained unchanged. All the results indicated that exposure to BPS altered the whole-body THs and TSH concentrations and changed the expression profiling of key genes related to HPT axis, thus triggering thyroid endocrine disruption.

## Introduction

Due to the significant adverse effects of bisphenol A (BPA) on human health, many regulatory agencies including Health Canada (2009), the US Food and Drug Administration (2010) and the European Union (EC regulation 321/2011) have decided to ban its use in the manufacture of polycarbonate infant feeding bottles. As a direct consequence, structural BPA analogues have been introduced to replace BPA in numerous commercial, industrial and consumer products during the past decade [1,2]. Bisphenol-S [bis-(4-hydroxyphenyl)-sulfone, (BPS)], which has rigid double bonds of the O = S = O group, is believed to have a higher thermal stability than BPA and is considered to be able to partially replace BPA [2]. This chemical is now widely used for a variety of industrial applications such as electroplating solvent, dyestuffs, color-fast agents, leather tanning agents, flame retardants, thermal receipt papers and fiber improvers [3]. According to European Chemicals Agency (2014), BPS is gradually replacing BPA with increasing production between 1000 and 10,000 t per year [4].

Considering its widespread application in various products, BPS has been detected in various environmental media, such as, indoor dust, surface water, sediment and sewage effluents [5,6]. For example, BPS has been detected in concentration ranges of 0.83 ng/g and 26.6 μg/g in indoor dust [7]. Yamazaki et al. [8] reported that BPS concentrations in water samples from Pearl River in China (135 ng/L) and Adyar River in India (6840 ng/L) were considerably higher than that of BPA (73 and 359 ng/L for the two rivers, respectively). Previous data also demonstrated that in sediments collected from the USA, Japan, and Korea, BPS has been detected in concentration ranges of 0.002–1970 ng/g dry weight (dw) [9], while in a municipal sewage sludge collected in 20 provinces of China, the detected concentrations were in the range of 0.17–110 ng/g dw [5]. In addition, BPS has been reported in food products in concentration ranges of 0.005–0.609 ng/g fresh weight (fw) in the USA [10], and up to 36.1± 4.4 ng/g fw in canned foods from Spain [11]. Consequently, BPS has also been detected in urine from the citizens of 8 countries and within the same concentration ranges as BPA [2,12].

Growing concern has been raised about the safety of BPS attribute to its structural similarity to BPA and widespread use. However, there is little information available on the toxic and/or endocrine-disrupting effects of BPS. Previous studies have demonstrated that BPS exerts estrogenic and anti-androgenic activity [13–16]. For example, results from *in vitro* assays have demonstrated that BPS similar to BPA, display comparable estrogenic activity, disrupt multiple nuclear receptors and may therefore interfered with the endocrine system in MELN cells [3,13]. *In vivo* assays, Naderi et al. [17] demonstrated that developmental exposure to BPS (≤ 100 μg/L) for 75 days caused developmental toxicity, disturbed the balance of hormones (sex steroids and thyroid hormones), and decreased egg production and sperm count in zebrafish. Similarly, exposure of adult zebrafish to low concentrations of BPS (≥0.5 μg/L) for 21 days significantly impaired normal reproduction and reduced gonadosomatic index in females, which affected the feedback regulatory circuits of hypothalamic-pituitary-gonad (HPG) axis and impaired the development of offspring [18]. These results further indicate that BPS has the potential to disrupt and interfere with the normal functions of endocrine system in organisms and may consequently lead to adverse outcomes.

In vertebrates, thyroid hormones (THs) play crucial roles in many physiological processes including growth, development, energy balance, metabolism, and reproduction action [19,20]. The hypothalamic-pituitary-thyroid (HPT) axis controls thyroid endocrine system which is responsible for the THs synthesis, secretion, transport and metabolism. Previous studies have indicated that the thyroid system of zebrafish is similar to mammalian or amphibian in many aspects, which can provide valuable information for human beings [21]. Moreover, the early life stages of fish is the most vulnerable to environmental pollutants, and developing zebrafish embryos/larvae is believed to be a reliable model to evaluate thyroid endocrine disruption by chemical treatment [22–24]. Therefore, in this study, we used zebrafish embryos/larvae to evaluate the HPT axis disturbance and explore the potential mechanisms after exposure to BPS.

## Materials and methods

### Materials and reagents

BPS (CAS No. 80-09-1) was purchased from Sigma-Aldrich (St. Louis, MO, USA). Stock solutions of BPS were prepared in dimethyl sulfoxide (DMSO) and stored in the dark at 4°C. Solvent concentration was kept at 0.01% DMSO (v/v) throughout the experiment. Methane-sulfonate salt (MS-222) was obtained from Sigma (St. Louis, MO, USA), which was used as an anesthetic to treat zebrafish larvae before sampling. All other chemicals used were analytical grade.

### Fish maintenance and experimental design

Adult zebrafish (*Danio rerio*) of the wild-type (AB strain) were maintained under an artificial light/dark period of 14/10 h and a constant temperature (28 ± 1°C) in an aerated aquarium system. Fish were fed twice daily with a commercial flake food (Tetramin, Germany) complemented with newly hatched brine shrimp (*Artemianauplii*). Fertilized eggs were obtained from spawning adults in tanks overnight and examined under a dissecting microscope. Embryos developing normally at blastula stage (30% epiboly) were selected for the subsequent experiments. Approximately 500 embryos were randomly distributed into each glass beakers containing 500 mL of BPS exposure solution (0, 1, 3, 10 and 30μg/L). The selected exposure concentrations were confirmed by a previous study [18] and based on the environmental concentration [8]. There were six replicates for each exposure concentration, and both exposure and control groups received 0.01% (v/v) DMSO. Zebrafish embryos/larvae were maintained in a biochemical incubator with 28°C and 14:10 (light: dark) condition. During the experimental period, the exposure solutions were renewed daily, and animal health monitoring was conducted simultaneously. The hatching rate of embryos at 72 hpf and 96 hpf were calculated. The larvae developmental parameters for body weight, body length (head-to tail axis), survival rate and malformation rate at 168 hpf were also recorded. After exposure to BPS until 168 h, zebrafish larvae were washed with UltraPure water and anesthetized in 0.03% MS-222 for random sampling. Then, the samples were flash-frozen in liquid nitrogen before storing at -80°C for the subsequent analysis of gene transcription, thyroid hormones and TSH assay. All fish were maintained in accordance with guidelines for the care and use of laboratory animals of the National Institute for Food and Drug Control of China. All animals were treated humanely and with the aim of alleviating any suffering.

### Ethics statement

This study was approved by the Institutional Animal Care and Use Committee (IACUC) of the Second Xiangya Hospital, Central South University, China.

### Chemical analysis of BPS

To measure actual concentrations of the exposure media, water samples were collected from each tank at 144 hpf and 168 hpf and stored at -20°C for the analysis of BPS concentrations. BPS concentration in the exposure solutions (n = 6 replicates) were extracted and performed according to previously published methods [6,25]. BPS concentrations were also determined in zebrafish larvae. Briefly, zebrafish larvae for each treatment were weighed before and after freeze-drying, crushed with 5 mL dichloromethane/methanol (2:1 v/v) by homogenizer, and then ultrasonic extraction for 5 min. A 0.9% KCl solution was added to the mixture (20% of final volume). After centrifugation at 1000 × g for 10 min, the dichloromethane phase was removed and evaporated to dryness and redissolved in 1 ml of methanol:hexane (1:20). Samples were applied to Sep-Pak NH_2_ 500-mg cartridges, previously conditioned with 5ml methanol and 5 ml methanol:hexane (1:20), at a flow of 1 ml /min. Before desorption, the cartridges were washed with 5 ml of hexane. After drying, samples were desorbed by 4 ml of methanol. Finally, the extract was evaporated to dryness using a flow of N_2_ and redissolved in 200 μL of methanol:H_2_O (3:2) for analysis. An API 4000 electrospray triple quadrupole mass spectrometer (ESI-MS/MS; Applied Biosystems, Foster City, CA, USA), coupled with a high-performance liquid chromatography (HPLC, Agilent 1100 Series, Agilent Technologies, Palo Alto, CA, USA) was used for identification and quantification of BPS. Procedural blanks and spiked blanks were analyzed simultaneously. The detection limit was defined as a signal/noise ratio (S/N) of 3.

### Thyroid hormones and TSH assay

Extraction of THs was carried out as described by Wang et al. [26]. Then, whole-body TH concentrations were measured using commercial ELISA kits (Uscnlife, Wuhan, China) based on the competitive binding enzyme immunoassay method. The limit of detection (LOD) for T3 and T4 were 0.1 and 1.2 ng/mL, respectively. Intra-assay and inter-assay variations were 4.5% and 7.2% for T3, and 4.3% and 7.5% for T4, respectively. No significant cross-reactivity or interference was observed for either kit. Likewise, whole-body TSH concentrations were measured using ELISA with a commercial kit for fish (CUSABIO, Wuhan, China) following the method described in the kit. Briefly, 200 zebrafish larvae were homogenized in 1 mL of PBS and then stored overnight at -20°C. Two freeze-thaw cycles were employed to break the cell membranes. Then, the homogenates were centrifuged at 5000 ×g for 5 min at 4°C, and 50 μL of the supernatant was used for the analysis of TSH. This commercial kit has been validated for the determination of TSH in zebrafish larvae previously [27]. The LOD for TSH was 2.5μIU/mL. Intra- and inter-assay were below 15% in this study.

### RNA isolation and quantitative real-time polymerase chainreaction (qRT-PCR)

Whole-body of zebrafish larvae were homogenized (30 larvae for each replicate, n = 6 replicates), and total RNA was extracted using Trizol reagents (Takara, Dalian, China) according to the manufacturer’s instructions. Total RNA concentration was estimated based on the results of NanoDrop 2000 (Thermo, Logan, UT, USA). The RNA quality was examined by measuring the 260/280 nm ratios and 1% agarose-formaldehyde gel electrophoresis with GoldView^™^ staining. Genomic DNA contamination was removed by using RNase-free DNase I (Promega, Madison, WI, USA). The synthesis of first-strand complementary DNA (cDNA) was performed by using M-MLV reverse transcriptase (Promega, Madison, WI, USA) with 2 μg RNA reverse-transcribed in each sample. Quantitative realtime polymerase chain reaction (qRT-PCR) was performed using SYBR^®^ Green Real-time PCR Master-Mix-Plus kits (Toyobo, Osaka, Japan) and analyzed on an ABI 7300 System (PerkinElmer Applied Biosystems, Foster City, CA, USA). The primer sequences of the selected genes were obtained using the online primer 3 program (http://frodo.wi.mit.edu/) and shown in Table 1. The PCR reaction cycle conditions used were: 50°C for1 min, 95°C for 10 min, followed by 40 cycles of 95°C for 15 s and60°C for 1 min.

**Table 1:** Primer sequences for tested genes.

Gene name	Sense primer (5’-3’)	Antisense prime (5’-3’)	Gene bank accession no.
*rpl8*	ttgttggtgttgttgctggt	ggatgctcaacagggttcat	NM_200713
*crh*	ttcgggaagtaaccacaagc	ctgcactctattcgccttcc	NM_001007379
*pax8*	gaagatcgcggagtacaagc	ctgcactttagtgcggatga	AF072549
*slc5a5*	ggtggcatgaaggctgtaat	gatacggcatccattgttgg	NM_001089391
*tg*	ccagccgaaaggatagagttg	atgctgccgtggaatagga	XM_001335283
*ttr*	cgggtggagtttgacacttt	gctcagaaggagagccagta	BC081488
*trα*	cgggtggagtttgacacttt	gctcagaaggagagccagta	NM_001005598.2
*trβ*	tgggagatgatacgggttgt	ataggtgccgatccaatgtc	NM_131340
*dio1*	Gttcaaacagcttgtcaaggact	agcaagcctctcctccaagtt	BC076008
*dio2*	gcataggcagtcgctcattt	tgtggtctctcatccaacca	NM_212789
*dio3*	gagaccgctgatcctcaacttc	tcgatgtacaccagcagagagt	NM_001177935.3
*ugt1ab*	ccaccaagtctttccgtgtt	Gcagtccttcacaggctttc	NM_213422

Following completion of each qRT-PCR reaction, melting curve analyses were performed to validate the specificity of the PCR amplifications. For quantification of PCR results, the threshold cycle (Ct) was determined for each reaction. The Ct values for each gene of interest were normalized to the endogenous control gene, ribosomal protein L8 (*rpl8*), by using the 2^–ΔΔCt^ method [28]. Using geNorm analysis, our studies demonstrated that *rpl8* was the most stable gene among the 5 commonly used housekeepinggenes (*β-actin*, *tubulin alpha 1* [*tuba1*], *18s rRNA* [*18S rRNA*], *elongation factor 1-alpha* [*elfa*], and *ribosomal proteinL8* [*rpl8*]) upon BPS exposure. Normalized values were used to calculate the degree of induction or inhibition, expressed as a fold difference compared with normalized control values.

### Statistical analysis

Normality of distribution and homogeneity of variance of each sample set were analyzed using the Kolmogorov–Smirnov test and Levene’s test, respectively. For hatching, survival and malformation rates, as these data were presented as proportions, they were square root arcsine-transformed before analysis of variance. All data are shown as means ± standard error(SEM), and the differences between the control and exposure group were analyzed by one-way analysis of variance (ANOVA), followed by Tukey's test. All the analyses were conducted with SPSS statistical software version 13.0 (SPSS, Inc., Chicago, IL, USA). A *P*< 0.05 was considered statistically significant.

## Results

### BPS in water and zebrafish larvae

**Fig 1:**
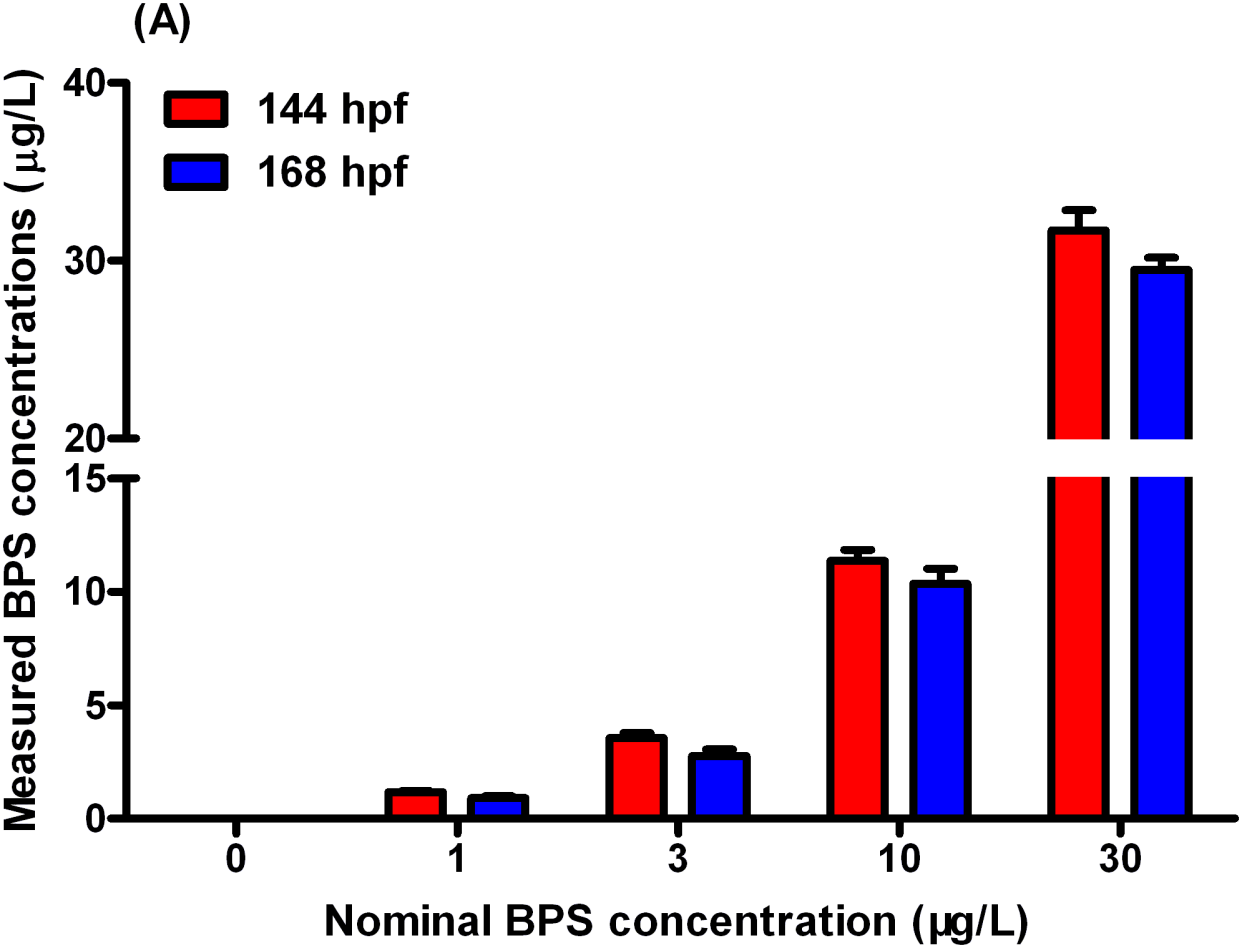
Concentrations of BPS in exposure solutions at 144 h post-fertilization (hpf) and 168 hpf. Values represent mean ± SEM (n = 6 samples).

In the water samples, concentrations of BPS in the solvent-control were below the detection limit. Mean concentrations of BPS at 144hpf were 1.18, 3.57, 11.36, and 33.70μg/Lin the 1-, 3-, 10-, and 30-μg/L exposure groups, respectively (Fig 1, Table A in S1 File, Data A in S1 Dataset). At 168hpf, mean concentrations of BPS in the exposure solutions were 0.93, 2.76, 10.36, and 29.50μg/L in the 1-, 3-, 10-, and 30-μg/L exposure groups, respectively (Fig 1, Table A in S1 File, Data A in S1 Dataset). For simplification, all results presented using the nominal concentrations.

**Fig 2:**
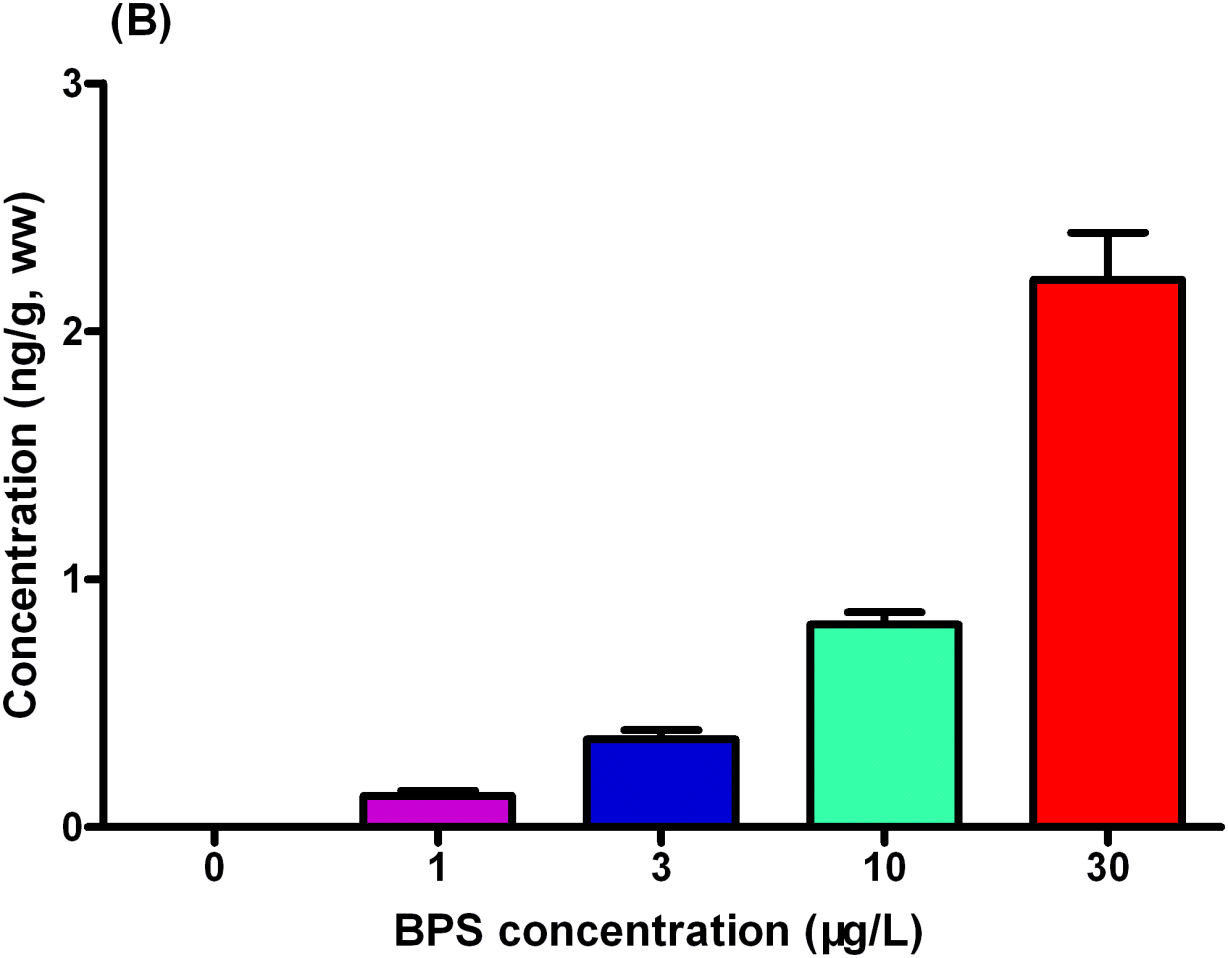
Concentrations of BPS in zebrafish larvae. Values represent mean ± SEM (n = 6 samples).

In the larvae, the detected BPS concentrations were 0.13, 0.36, 0.82, and 2.21 ng/g wet weight in the 1-, 3-, 10, and 30 μg/L exposure groups, respectively (Fig 2, Table B in S1 File, Data B in S1 Dataset).

### Developmental parameters of zebrafish larvae

Exposure to BPS did not affect embryo survival or growth parameters (body weight and length) at 168 hpf (Table 2). No significant difference in the malformation rate was recorded in the exposure groups compared with the control group (Table 2). However, the hatching rate was significantly reduced in 30 μg/L exposure group compared with the corresponding control at 72 hpf, whereas the hatching rate was restored to levels similar to control levels at 96 hpf (>90%)(Table 2).

**Table 2:** Survival, malformation, body length, weight, and hatchability of zebrafish larvae after exposure to BPS (0, 1, 3, 10, and 30 μg/L).

BPS	0	1	3	10	30
168hpf Survival (%)	86.4±2.03	88.1±4.36	85.7±4.91	83.6±3.84	85.2±5.51
168hpf Weight (mg)	0.4 ± 0.02	0.4 ± 0.04	0.43 ± 0.05	0.38 ± 0.01	0.37 ± 0.03
168hpf Length (mm)	3.73 ± 0.15	3.50 ± 0.23	3.63 ± 0.27	3.47 ± 0.38	3.80 ± 0.17
168 hpf Malformation (%)	3.35 ± 0.88	3.02 ± 1.53	3.34 ± 1.45	3.67 ± 0.88	3.96 ± 1.20
72 hpf Hatchability (%)	81.2 ± 2.65	79.5 ± 4.04	73.3 ± 3.76	74.1 ± 3.2	62.3 ± 5.55*
96 hpf Hatchability (%)	94.5 ± 1.2	93.3 ± 1.0	90.8 ± 1.4	91.2 ± 1.7	90.8 ± 1.9

The values represent as mean ± standard error (SEM) of six replicate groups. Asterisk indicates significant difference compared with solvent control (* p < 0.05).

### mRNA expression profiles

**Fig 3:**
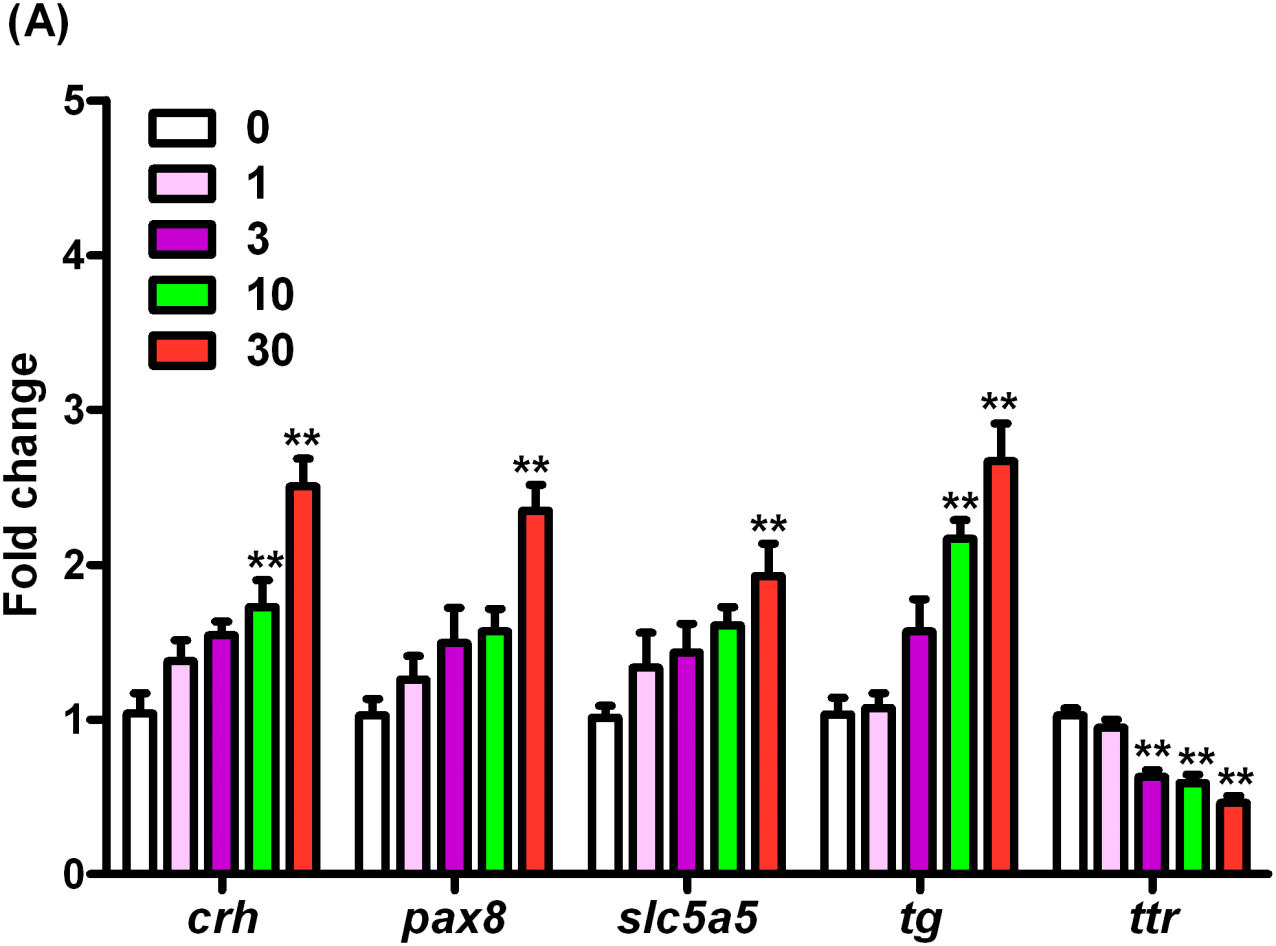
mRNA expression of crh, pax8, slc5a5, tg, and ttr in168-hpf zebrafish embryos/larvae exposed to BPS. Values represent mean ± SEM (n = 6 samples; each sample included 30 larvae). Significant difference from the control group is indicated by *P < 0.05, **P < 0.01.

The transcription of *crh* gene was significantly induced by 1.66- and 2.41-fold in a dose dependent manner after exposure to10 and 30 μg/L of BPS as compared to that in the control, respectively (Fig 3, Table C in S1 File, Data C in S1 Dataset). After exposure to 30 μg/L of BPS, the transcriptional levels of *pax8* and *slc5a5* were significantly up-regulated by 2.28- and 1.90-fold, respectively (Fig 3, Table C in S1 File, Data C in S1 Dataset). The mRNA expression of gene encoding *tg* was transcriptionally up-regulated by 2.09- and 2.58-fold upon treatment with 10 and 30 μg/L of BPS, respectively (Fig 3, Table C in S1 File, Data C in S1 Dataset). However, the *ttr* gene was significantly inhibited by 1.63-, 1.74-, and 2.22-fold at a concentration-dependent manner after exposure to 3, 10, and 30 μg/L of BPS, respectively (Fig 3, Table C in S1 File, Data C in S1 Dataset).

**Fig 4:**
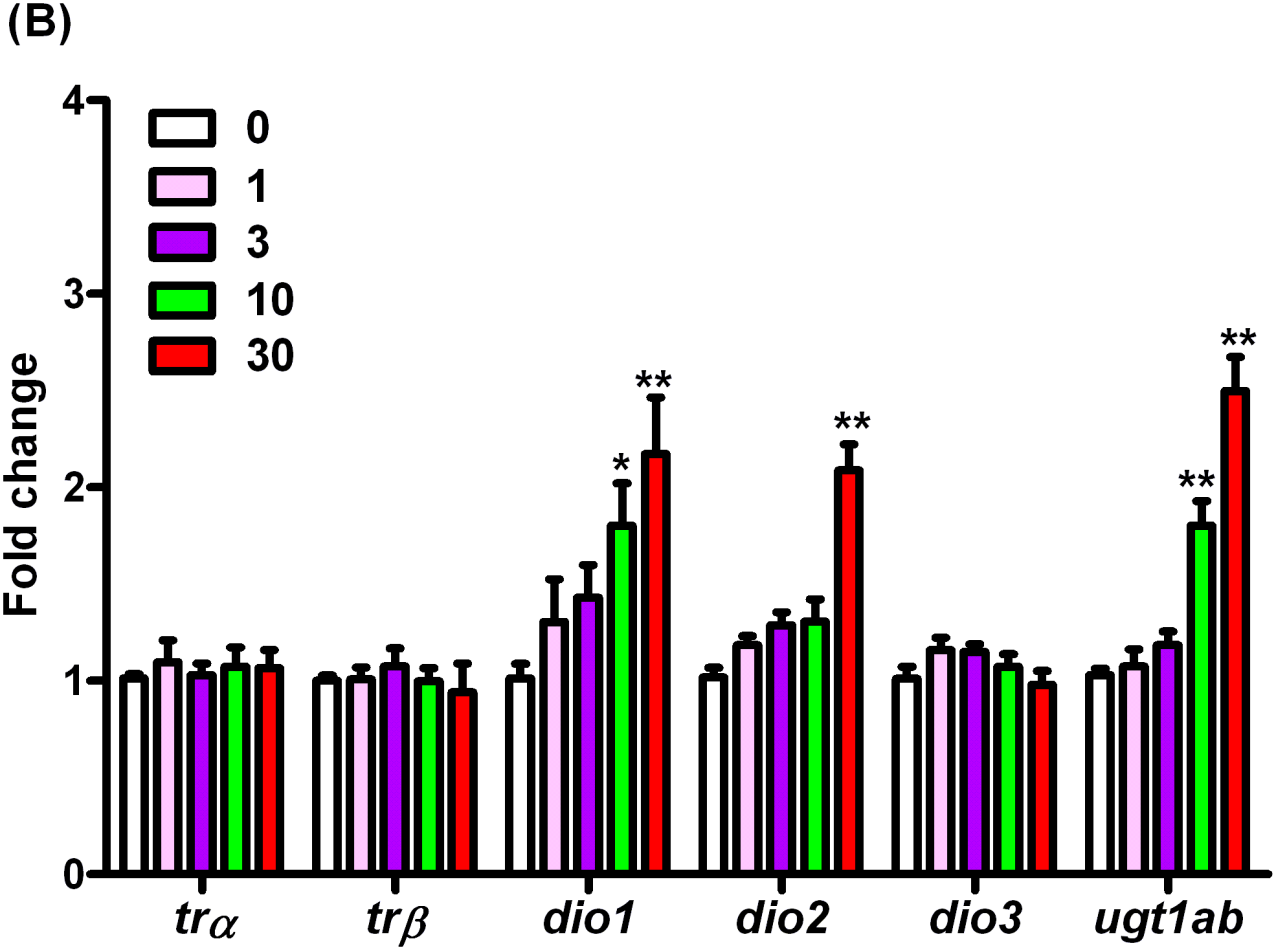
mRNA expression of trα, trβ, dio1, dio2, dio3, and ugt1ab in168-hpf zebrafish embryos/larvae exposed to BPS. Values represent mean ± SEM (n = 6 samples; each sample included 30 larvae). Significant difference from the control group is indicated by *P < 0.05, **P < 0.01.

In this study, three deiodinase isoforms (Dio1, Dio2 and Dio3) have been examined. The transcription of*dio1* gene was significantly up-regulated by 1.78- and 2.14-fold after exposure to 10 and 30 μg/L of BPS, respectively. The *dio2* was increased significantly by 2.05-fold after exposure to 30 μg/L of BPS, while no significant difference was observed in *dio3* gene (Fig 4, Table D in S1 File, Data D in S1 Dataset). In addition, mRNA level of *ugt1ab*involved in the metabolism of TH was significantly induced by 1.75- and 2.43-fold after exposure to 10 and 30 μg/L of BPS, respectively (Fig 4, Table D in S1 File, Data D in S1 Dataset). However, the expressions of thyroid hormone nuclear receptors isoforms, trα and trβ showed no significant alteration in zebrafish after exposure to 0, 1, 3, 10, and 30 μg/L of BPS for 7 days, respectively (Fig 4, Table D in S1 File, Data D in S1 Dataset).

### Whole-body T4 and T3 contents

**Fig 5:**
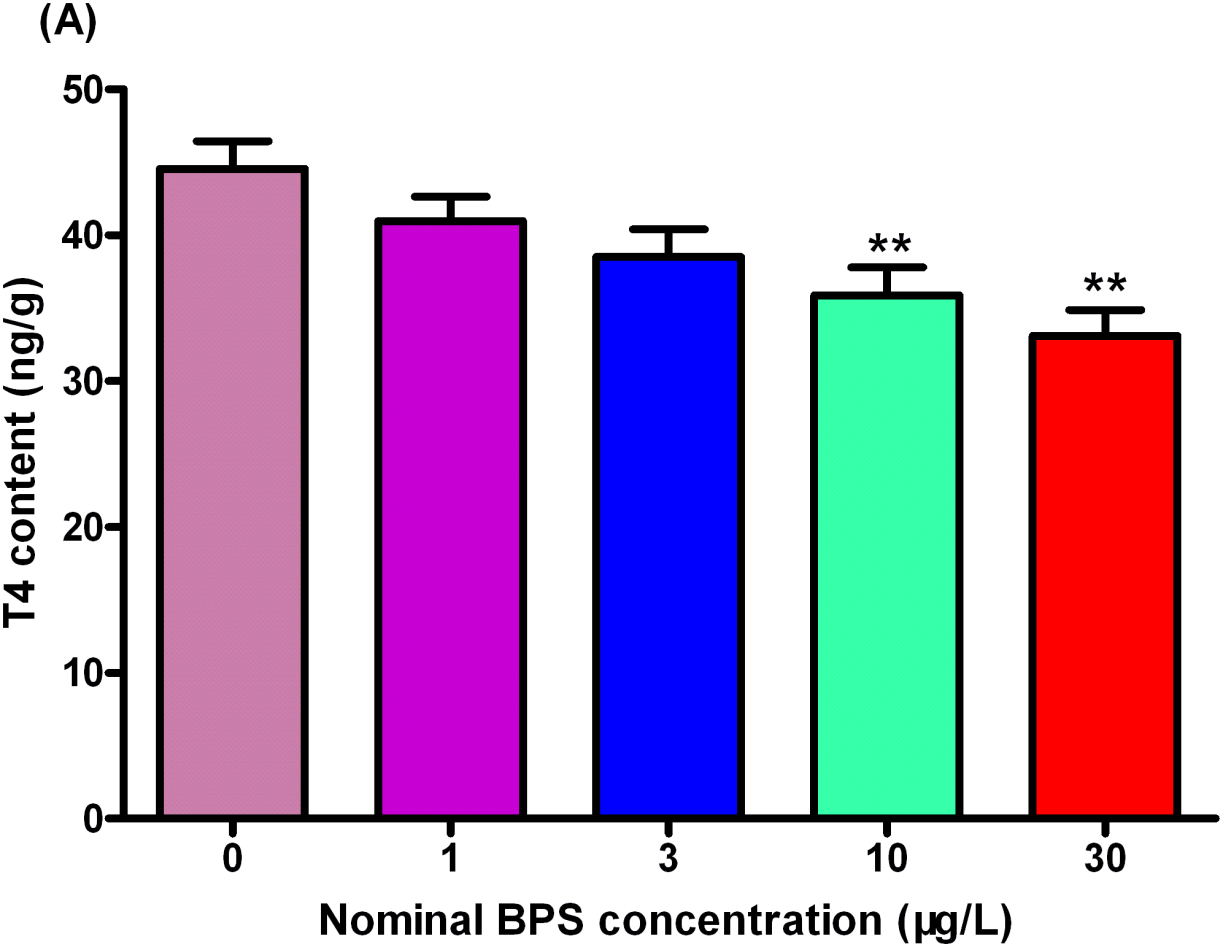
Whole-body thyroxine (T4) contents in zebrafish larvae exposed to different concentrations of BPS. Values represent mean ± SEM (n = 6). Significant differences between BPS exposure groups and the corresponding control group are indicated by *P < 0.05 and **P < 0.01.

As shown in Fig 5, BPS exposure caused a dose-dependent decrease in whole-body T4 concentrations. Compared to the control groups, the T4 concentrations were significantly decreased by 19.5% and 25.7% at 10 and 30 μg/L BPA-treated groups, respectively (Fig 5, Table E in S1 File, Data E in S1 Dataset).

**Fig 6:**
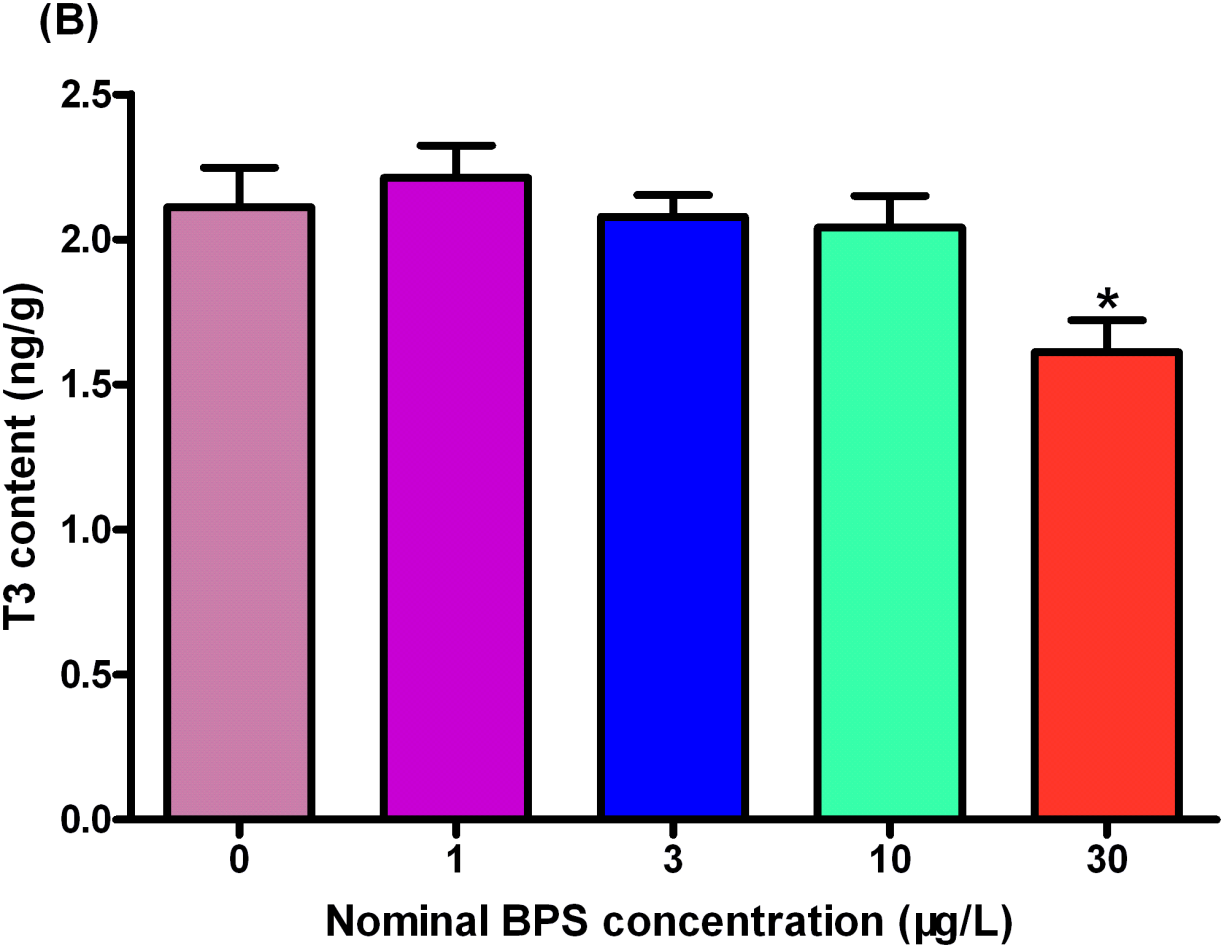
Whole-body triiodothyronine (T3) contents in zebrafish larvae exposed to different concentrations of BPS. Values represent mean ± SEM (n = 6). Significant differences between BPS exposure groups and the corresponding control group are indicated by *P < 0.05 and **P < 0.01.

Moreover, the concentrations of whole-body T3 in tissue homogenate were significantly inhibited by 23.7% at 30 μg/L exposure group as compared to that in the control (Fig 6, Table F in S1 File, Data F in S1 Dataset).

### Whole-body TSH content

**Fig 7:**
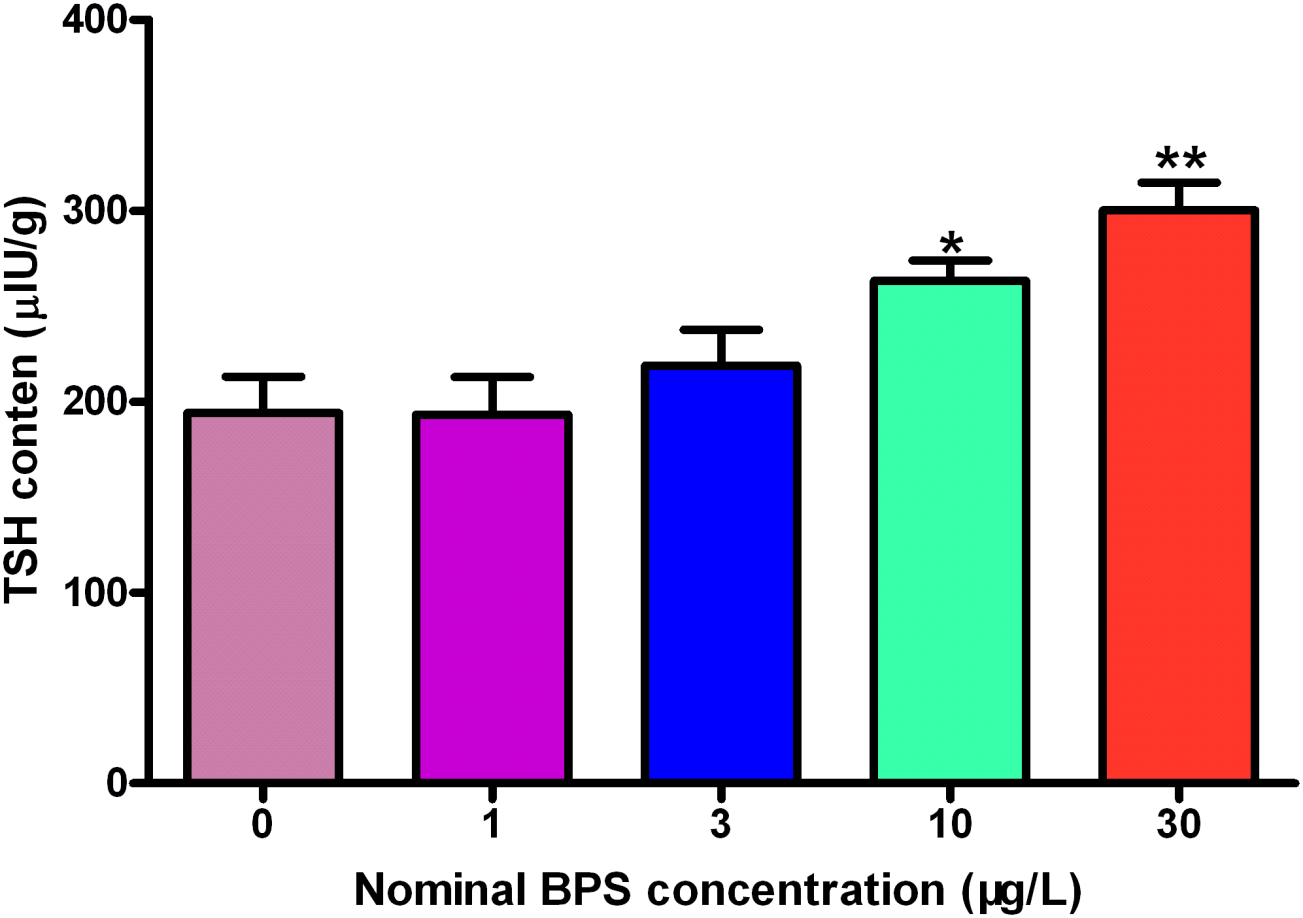
Whole-body thyroid-stimulating hormone (TSH) contents in zebrafish larvae exposed to different concentrations of BPS. Values represent mean ± SEM (n = 6). Significant differences between BPS exposure groups and the corresponding control group are indicated by *P < 0.05 and **P < 0.01.

The TSH content of the whole body in the control group was determined to be 194 μIU/g (Fig 7, Table G in S1 File, Data G in S1 Dataset). There was no significant difference in the TSH contents of the embryos exposed to 1 and 3 μg/L of BPS and those in the control group (Fig 7, Table G in S1 File, Data G in S1 Dataset). However, TSH contents were significantly increase by 35.6% and 54.6% in the 10-, and 30 μg/L exposure groups compared with those in the control group, respectively (Fig 7, S Table G in S1 File, Data G in S1 Dataset).

## Discussion

The pollutants of BPA and its analogs have gained great concern because of their potentially adverse health impacts. BPS has emerged as a potential BPA replacement alternative, which has a higher thermal stability than BPA and its production is rising annually [29]. Growing evidence have suggested that BPS has the potential to disrupt the normal functions of endocrine system, such as triggers reproductive toxicity and disturbs hormonal balance in different animal models [17,30]. In this study, we used zebrafish embryos/larvae as the model to evaluate the mechanism of thyroid endocrine disruption caused by BPS. The results demonstrated that BPS significantly changed whole-body TH and TSH concentrations as well as modified the mRNA expression of key genes involved in HPT axis, suggesting the thyroid endocrine disrupting effects of BPS in zebrafish embryos/larvae.

In the present study, we observed a significant decrease in hatchability at 72 hpf in the 30 μg/L of BPS treated groups. Hatching delay was also reported in previous studies after exposure to other bisphenols. For example, Shi et al. [31] demonstrated that developmental exposure of zebrafish embryos to BPAF (≥5 μg/L) from 4 hpf to 120 day-post-fertilization (dpf) caused significant hatching delay in the F1 generation at 72 hpf. Similarly, parental exposure to BPS adversely affected hatching rate and time to hatching of the offspring even when they were hatched in clean water [17,18]. In addition, significantly delayed hatchability at 72 hpf was observed in zebrafish embryos after waterborne exposure to 50 and 500 μg/L of BPAF [32], which is consistent with the results of our study.

In fish, thyroid hormones are crucial for the early development, especially during the larval development stage. In this study, the levels of whole-body T4 and T3 concentrations were significantly reduced after exposure to BPS, indicating BPS could cause hypothyroidism in zebrafish larvae. This result is in agreement with previous report, which demonstrated that development exposure of zebrafish to BPS for 75 days significantly decreased plasma T4 and T3 levels in both sexes [17]. Disturbance of THs concentrations by other bisphenols have also been reported in previous studies. For instance, waterborne exposure of zebrafish embryos to bisphenol F (BPF) or bisphenol AF (BPAF) significantly altered THs concentrations as well as gene transcription involved in the HPT axis [27,32].

It has been suggested that the pituitary gland secreted TSH to regulate TH synthesis and release from the thyroid gland [33]. Thus, TSH assay can be used to evaluate the functional integrity of the pituitary and thyroid gland, and explore the potential mechanism of chemical disruption of thyroid dysfunction [34]. The present data demonstrated that TSH contents were significantly induced in a concentration-dependent manner after BPS exposure, which might involve in negative feedback mechanism. Likewise, reductions in T4 concentrations accompanied with increase of TSH contents have been reported in zebrafish larvae after exposure to BPF [27]. Moreover, previous studies have indicated that corticotropin-releasing hormone (CRH) appears to stimulate TSH secretion [35], and might function as a common regulator of the HPT axis in fish. In this study, transcription of *crh* gene was significantly upregulated after BPS treatment. Hence, we speculated that the upregulation of *crh* and increased TSH contents may promote TH synthesis and release to compensate for the decreased T4 levels in zebrafish larvae. Similar results have also been observed for zebrafish larvae after exposure to various pollutants [36,37].

Previous studies demonstrated that the products of *slc5a5* and *tg* are involved in TH synthesis [34], and Pax8 protein is essential for the late differentiation of the follicular cells [38]. Moreover, as a thyroid transcription factor, *pax8* regulates the transcription of *slc5a5* and *tg* genes in vertebrate [34,39]. In this study, the mRNA expression of *pax8*, *slc5a5* and *tg* was noted to be significantly increased after exposure to BPS. Increase of *pax8* concomitant upregulated *slc5a5* and *tg* genes have also been reported for zebrafish embryos/larvae exposed to BPF, or other xenobiotics [26,27,36]. Combined with these results, we speculated that the up-regulation of *pax8* might promote the increase of *slc5a5* and *tg* expression as a negative feedback mechanism for the regulation of the lowered T4 levels.

In fish, transthyretin (TTR) is a major transport protein and plays an important role in regulating the supply of THs to various target tissues [40,41]. The combined T4 would make it more stable to hepatic metabolism, resulting in a lower clearance and an increase in circulating TH concentrations [41]. Therefore, chemicals interfering with the binding of THs to TTR may directly affect free TH levels and its clearance rate in fish. Previous studies demonstrated that decrease of *ttr* mRNA level was coincident with the reduction ofT4levels after chemical treatment [36,42]. In the present study, *ttr* gene transcription decreased significantly after exposure to BPS, indicating that BPS posed a potential risk to thyroid functions by affecting the binding and transporting of free THs.

Three types of deiodinases enzymes (Dio1, Dio2 and Dio3) have been identified in fish, and each of them plays different roles in the regulation of TH levels. Studies proved that Dio1 has a major influence on iodine recovery and TH removal [43], while Dio2 catalyzed T4 to biological active T3 in the peripheral tissues, and Dio3 is a purely inactivating enzyme[44]. In this study, *dio1* and *dio2* mRNA levels were increased significantly after BPS exposure at ≥ 10 and 30 μg/L, respectively, while the expression of *dio3* remained unchanged. It was demonstrated that hypothyroidism was related to the increase of *dio2* and/or *dio1* mRNA expression, when zebrafish larvae were exposed to other environmental pollutants [26,27,32,37]. Thus, our results suggested that BPS as an environment endocrine disrupting chemical decreased THs content and upregulated *dio1* and *dio2* expression via HPT axis in the zebrafish larvae.

It was indicated that uridinediphosphate glucuronosyltransferase (UGT) played important role in TH homeostasis by the T4 conjugation pathway [45]. For instance, Barter et al. [46,47] demonstrated that exposure to UGT inducer significantly decreased T4 concentrations in the plasma of rats. Other studies also demonstrated that the lowered T4 content was accompanied with the increased *ugt1ab* expression after exposure to environmental pollutants on zebrafish larvae [26,27,48]. In the present study, we observed significant induction of *ugt1ab* expression at ≥ 10 μg/L when exposed to BPS. Therefore, we proposed that the increase of *ugt1ab* may be another reason for the decrease of THs content in the zebrafish larvae.

In fish, the biological functions of THs are proposed to be mediated by binding to thyroid hormone receptors (TRs) [49], and previous study demonstrated that TRs could affect the development of embryos [50]. In the present study, we observed no alteration of TRs expression and no significant effects on the development of zebrafish larvae after exposure to BPS. Similar result was also reported in previous study [51].

Previous study demonstrated that a supra-environmental dose of BPA in the water (1 μM) results in an environmentally relevant dose in larval zebrafish (10 μg/kg) [52]. However, in the present study, BPS was observed in the developing larvae with higher bioconcentration factor (BCF). This difference may be attributed to longer exposure duration employed in this study. It should also be noted that the measured BPS content in the zebrafish larvae from the exposure groups were similar to those reported in foodstuffs from the United States [10], demonstrating its health risk for human exposure. In conclusion, this study demonstrated that exposure to BPS altered THs and TSH levels by modulating the expression of HPT axis related genes, proving the BPS toxicity on thyroid endocrine system in zebrafish larvae. As an alternative of BPA, other toxicological effects such as organ development (e.g. otolith, which has been proven to be targeted by BPA and other bisphenols [53]) should be paid attention in future studies. In addition, given the fact that BPS as well as other bisphenols is widely used in daily life and inevitably released into the natural environment, more attention should be paid to evaluating the potential risks caused by chronic exposure to bisphenols on aquatic organisms and human beings.

## Supporting information

S1 Dataset **Data A:** Concentrations of BPS in exposure solutions. **Data B:** BPS content in zebrafish larvae. **Data C:** mRNA expression of *crh*, *pax8*, *slc5a5*, *tg*, and *ttr*. **Data D:** mRNA expression of *trα*, *trβ*, *dio1*, *dio2*, *dio3*, and *ugt1ab*. **Data E:** Whole-body T4 contents. **Data F:** Whole-body T3 contents. **Data G:** Whole-body TSH contents.
(XLSX)

S1 File **Table A:** Concentrations of BPS in exposure solutions. **Table B:** BPS content in zebrafish larvae. **Table C:** mRNA expression of *crh*, *pax8*, *slc5a5*, *tg*, and *ttr*. **Table D:** mRNA expression of *trα*, *trβ*, *dio1*, *dio2*, *dio3*, and *ugt1ab*. **Table E:** Whole-body T4 contents. **Table F:** Whole-body T3 contents. **Table G:** Whole-body TSH contents.
(DOCX)
