# Supplementary material for: Waterborne exposure to BPS causes thyroid endocrine disruption in zebrafish larvae
Source: PLoS One. 2017 May 3;12(5):e0176927. doi: 10.1371/journal.pone.0176927 (PMC5415059; doi:10.1371/journal.pone.0176927)
Supplement: S1 File — Table A: Concentrations of BPS in exposure solutions. Table B: BPS content in zebrafish larvae. Table C: mRNA expression of crh, pax8, slc5a5, tg, and ttr. Table D: mRNA expression of trα, trβ, dio1, dio2, dio3, and ugt1ab. Table E: Whole-body T4 contents. Table F: Whole-body T3 contents. Table G: Whole-body TSH contents. (DOCX) [file pone.0176927.s002.docx]

**Table A.** Concentrations of BPS in exposure solutions at 144 h post-fertilization (hpf) and 168 hpf.

|  | 144 hpf |  |  |  | 168 hpf |  |  |
| --- | --- | --- | --- | --- | --- | --- | --- |
| BPS (μg/L) | Mean (SEM) | Median (SD) | Q1 (Q3) |  | Mean (SEM) | Median (SD) | Q1 (Q3) |
| 0 | 0.0 (0.0) | 0.0 (0.0) | 0.0 (0.0) |  | 0.0 (0.0) | 0.0 (0.0) | 0.0 (0.0) |
| 1 | 1.18 (0.06) | 1.16 (0.16) | 1.03 (1.34) |  | 0.93 (0.08) | 0.92 (0.20) | 0.78 (1.09) |
| 3 | 3.57 (0.20) | 3.48 (0.50) | 3.11 (4.12) |  | 2.76 (0.31) | 2.53 (0.75) | 2.25 (3.42) |
| 10 | 11.36 (0.49) | 11.35 (1.19) | 10.39 (12.18) |  | 10.36 (0.65) | 10.01 (1.60) | 9.35 (11.36) |
| 30 | 31.70 (1.14) | 32.50 (2.79) | 30.13 (33.73) |  | 29.50 (0.66) | 29.40 (1.63) | 28.13 (30.63) |

**Table B.** BPS content in zebrafish larvae.

|  |  |  |  |
| --- | --- | --- | --- |
| BPS (μg/L) | Mean (SEM) | Median (SD) | Q1 (Q3) |
| 0 | 0.0 (0.0) | 0.0 (0.0) | 0.0 (0.0) |
| 1 | 0.13 (0.02) | 0.13 (0.05) | 0.08 (0.17) |
| 3 | 0.36 (0.04) | 0.33 (0.09) | 0.28 (0.45) |
| 10 | 0.82 (0.05) | 0.81 (0.12) | 0.70 (0.95) |
| 30 | 2.21 (0.19) | 2.04 (0.47) | 1.93 (2.47) |

**Table C. mRNA expression of crh, pax8, slc5a5, tg, and ttr.**

|  | crh |  |  |  | pax8 |  |  |  | Slc5a5 |  |  |
| --- | --- | --- | --- | --- | --- | --- | --- | --- | --- | --- | --- |
| BPS  (μg/L) | Mean (SEM) | Median (SD) | Q1 (Q3) |  | Mean (SEM) | Median (SD) | Q1 (Q3) |  | Mean (SEM) | Median (SD) | Q1 (Q3) |
| 0 | 1.04 (0.13) | 0.96 (0.32) | 0.76 (1.33) |  | 1.03 (0.10) | 1.06 (0.25) | 0.83 (1.24) |  | 1.01 (0.08) | 0.93 (0.19) | 0.86 (1.23) |
| 1 | 1.38 (0.13) | 1.29 (0.33) | 1.07 (1.77) |  | 1.26 (0.15) | 1.20 (0.38) | 0.88 (1.70) |  | 1.33 (0.23) | 1.19 (0.55) | 0.96 (1.61) |
| 3 | 1.55 (0.09) | 1.53 (0.22) | 1.39 (1.71) |  | 1.50 (0.23) | 1.50 (0.55) | 0.95 (1.99) |  | 1.43 (0.19) | 1.43 (0.46) | 1.02 (1.82) |
| 10 | 1.73 (0.17) | 1.80 (0.42) | 1.25 (2.09) |  | 1.57 (0.15) | 1.54 (0.36) | 1.25 (1.88) |  | 1.61 (0.12) | 1.59 (0.30) | 1.34 (1.88) |
| 30 | 2.50 (0.18) | 2.45 (0.44) | 2.15 (2.70) |  | 2.35 (0.17) | 2.39 (0.41) | 1.89 (2.71) |  | 1.93 (0.21) | 2.01 (0.51) | 1.34 (2.39) |
|  |  |  |  |  |  |  |  |  |  |  |  |
|  | tg |  |  |  | ttr |  |  |  |  |  |  |
| 0 | 1.03 (0.11) | 0.95 (0.26) | 0.84 (1.25) |  | 1.03 (0.04) | 1.03 (0.11) | 0.94 (1.12) |  |  |  |  |
| 1 | 1.08 (0.09) | 1.02 (0.23) | 0.88 (1.35) |  | 0.95 (0.05) | 0.97 (0.13) | 0.88 (1.02) |  |  |  |  |
| 3 | 1.57 (0.21) | 1.34 (0.52) | 1.14 (2.20) |  | 0.63 (0.04) | 0.61 (0.11) | 0.57 (0.70) |  |  |  |  |
| 10 | 2.17 (0.13) | 2.10 (0.31) | 1.95 (2.29) |  | 0.59 (0.05) | 0.61 (0.13) | 0.47 (0.69) |  |  |  |  |
| 30 | 2.67 (0.24) | 2.71 (0.59) | 2.34 (3.20) |  | 0.46 (0.05) | 0.48 (0.11) | 0.34 (0.56) |  |  |  |  |

**Table D. mRNA expression of trα, trβ, dio1, dio2, dio3, and ugt1ab.**

|  | trα |  |  |  | trβ |  |  |  | dio1 |  |  |
| --- | --- | --- | --- | --- | --- | --- | --- | --- | --- | --- | --- |
| BPS  (μg/L) | Mean (SEM) | Median (SD) | Q1 (Q3) |  | Mean (SEM) | Median (SD) | Q1 (Q3) |  | Mean (SEM) | Median (SD) | Q1 (Q3) |
| 0 | 1.01 (0.02) | 1.01 (0.05) | 0.97 (1.06) |  | 1.00 (0.03) | 1.00 (0.06) | 0.96 (1.04) |  | 1.01 (0.07) | 1.09 (0.18) | 0.79 (1.15) |
| 1 | 1.10 (0.11) | 1.00 (0.27) | 0.89 (1.39) |  | 1.01 (0.06) | 1.03 (0.15) | 0.85 (1.13) |  | 1.31 (0.22) | 1.21 (0.54) | 0.88 (1.74) |
| 3 | 1.03 (0.06) | 1.06 (0.15) | 0.89 (1.11) |  | 1.07 (0.09) | 1.07 (0.23) | 0.87 (1.31) |  | 1.43 (0.17) | 1.46 (0.42) | 1.00 (1.84) |
| 10 | 1.07 (0.10) | 1.02 (0.25) | 0.89 (1.29) |  | 1.00 (0.07) | 1.00 (0.16) | 1.86 (1.12) |  | 1.80 (0.22) | 1.75 (0.53) | 1.24 (2.36) |
| 30 | 1.07 (0.09) | 0.97 (0.23) | 0.91 (1.27) |  | 0.94 (0.15) | 0.85 (0.36) | 0.66 (0.19) |  | 2.17 (0.29) | 2.06 (0.72) | 1.61 (2.87) |
|  |  |  |  |  |  |  |  |  |  |  |  |
|  | dio2 |  |  |  | dio3 |  |  |  | ugt1ab |  |  |
| 0 | 1.02 (0.05) | 1.03 (0.12) | 0.90 (1.12) |  | 1.01 (0.06) | 1.00 (0.15) | 0.91 (1.11) |  | 1.03 (0.03) | 1.05 (0.08) | 0.94 (1.09) |
| 1 | 1.19 (0.04) | 1.14 (0.11) | 1.11 (1.28) |  | 1.16 (0.07) | 1.11 (0.16) | 1.02 (1.31) |  | 1.08 (0.09) | 1.04 (0.22) | 0.90 (1.32) |
| 3 | 1.29 (0.04) | 1.23 (0.16) | 1.16 (1.48) |  | 1.15 (0.04) | 1.13 (0.10) | 1.08 (1.20) |  | 1.19 (0.07) | 1.14 (0.17) | 1.06 (1.36) |
| 10 | 1.31 (0.11) | 1.21 (0.28) | 1.16 (1.40) |  | 1.07 (0.06) | 1.05 (0.16) | 0.93 (1.26) |  | 1.80 (0.13) | 1.75 (0.31) | 1.55 (2.14) |
| 30 | 2.09 (0.14) | 1.99 (0.33) | 1.82 (2.37) |  | 0.98 (0.07) | 0.98 (0.17) | 0.83 (1.15) |  | 2.50 (0.17) | 2.43 (0.43) | 2.20 (2.84) |

**Table E. Whole-body T4 contents.**

|  | T4 |  |  |
| --- | --- | --- | --- |
| BPS  (μg/L) | Mean (SEM) | Median (SD) | Q1 (Q3) |
| 0 | 44.53 (1.88) | 44.04 (4.62) | 40.98 (47.81) |
| 1 | 40.97 (1.66) | 40.32 (4.07) | 37.36 (45.17) |
| 3 | 38.49 (1.89) | 37.69 (4.63) | 34.97 (42.02) |
| 10 | 35.86 (1.93) | 36.37 (4.73) | 30.93 (40.03) |
| 30 | 33.10 (1.76) | 32.13 (4.31) | 29.92 (36.58) |

**Table F. Whole-body T3 contents.**

|  | T3 |  |  |
| --- | --- | --- | --- |
| BPS  (μg/L) | Mean (SEM) | Median (SD) | Q1 (Q3) |
| 0 | 2.11 (0.14) | 2.20 (0.33) | 1.85 (2.34) |
| 1 | 2.21 (0.11) | 2.13 (0.27) | 2.05 (2.39) |
| 3 | 2.08 (0.08) | 2.10 (0.19) | 1.88 (2.21) |
| 10 | 2.04 (0.11) | 2.13 (0.27) | 1.72 (2.23) |
| 30 | 1.61 (0.11) | 1.64 (0.27) | 1.41 (1.82) |

**S7 G.** **Whole-body TSH content.**

|  | TSH |  |  |
| --- | --- | --- | --- |
| BPS  (μg/L) | Mean (SEM) | Median (SD) | Q1 (Q3) |
| 0 | 194.10 (18.84) | 181.90 (46.16) | 152.00 (241.40) |
| 1 | 193.10 (19.65) | 194.90 (48.16) | 146.80 (240.80) |
| 3 | 218.70 (18.95) | 229.60 (46.41) | 173.70 (253.90) |
| 10 | 263.30 (10.44) | 261.60 (25.56) | 240.50 (284.50) |
| 30 | 300.10 (14.70) | 287.70 (36.00) | 273.90 (328.10) |
